# Glioprotective Effects of Ashwagandha Leaf Extract against Lead Induced Toxicity

**DOI:** 10.1155/2014/182029

**Published:** 2014-05-28

**Authors:** Praveen Kumar, Raghavendra Singh, Arshed Nazmi, Dinesh Lakhanpal, Hardeep Kataria, Gurcharan Kaur

**Affiliations:** Department of Biotechnology, Guru Nanak Dev University, Amritsar, Punjab 143005, India

## Abstract

*Withania somnifera* (Ashwagandha), also known as Indian Ginseng, is a well-known Indian medicinal plant due to its antioxidative, antistress, antigenotoxic, and immunomodulatory properties. The present study was designed to assess and establish the cytoprotective potential of Ashwagandha leaf aqueous extract against lead induced toxicity. Pretreatment of C6 cells with 0.1% Ashwagandha extract showed cytoprotection against 25 **μ**M to 400 **μ**M concentration of lead nitrate. Further pretreatment with Ashwagandha extract to lead nitrate exposed cells (200 **μ**M) resulted in normalization of glial fibrillary acidic protein (GFAP) expression as well as heat shock protein (HSP70), mortalin, and neural cell adhesion molecule (NCAM) expression. Further, the cytoprotective efficacy of Ashwagandha extract was studied *in vivo*. Administration of Ashwagandha extract provided significant protection to lead induced altered antioxidant defense that may significantly compromise normal cellular function. Ashwagandha also provided a significant protection to lipid peroxidation (LPx) levels, catalase, and superoxide dismutase (SOD) but not reduced glutathione (GSH) contents in brain tissue as well as peripheral organs, liver and kidney, suggesting its ability to act as a free radical scavenger protecting cells against toxic insult. These results, thus, suggest that Ashwagandha water extract may have the potential therapeutic implication against lead poisoning.

## 1. Introduction

There is a growing interest in the use of herbal plants for their different medicinal properties due to their natural origin, cost effectiveness, and negligible side effects [1].* Withania somnifera* (Ashwagandha) is very popular in traditional Indian medicine system, Ayurveda. It is considered to be Indian Ginseng due to its rejuvenating effects on the body such as antioxidative, antistress, antigenotoxic, and immunomodulatory properties [2, 3]. Among the herbs classified as brain tonics or rejuvenators in the traditional Indian medicine system, Ashwagandha is the most important plant whose extracts make a significant component to the daily supplements for body and brain health. Although a variety of Ashwagandha extracts have displayed neuroprotective, neuroregenerative, and anticancer potentials in recent* in vitro* studies [4–7] using brain-derived cells, potentials of water extract of leaves of Ashwagandha (ASH-WEX) remain largely unexplored.

Lead, although one of the most useful metals, is also one of the most toxic environmental pollutant which is detected in almost all phases of biological systems. The mechanisms of lead induced neurotoxicity are complex. Oxidative stress, membrane biophysics alterations, deregulation of cell signalling, and the impairment of neurotransmission are considered as key aspects involved in lead neurotoxicity. The mechanism involves toxicity by oxidative stress [8] and free radical damage via two separate, albeit related, pathways: (a) the generation of reactive oxygen species (ROS), including hydroperoxides, singlet oxygen, and hydrogen peroxide, and (b) the direct depletion of antioxidant reserves [9].

Astroglial cells are the most abundant cells in the CNS and are believed to play a key role in the brain and spinal cord pathologies. Furthermore, it is established that glial cells and their resident protein GFAP integrate neuronal signals and modulate synaptic activity by formation of cytoskeletal filaments [10]. GFAP is the cytoskeletal protein of astrocytes which is involved in controlling movement and shape of astrocytes, differentiation marker of glial cells/and increased GFAP expression which has been associated with aging [11, 12]. Astrocytes are important target of lead toxicity and take up lead to store it intracellularly and, by sequestering lead, astrocytes may protect more sensitive neurons [13]. It is well established on getting exposed to stress; the glial cells react by upregulating GFAP expression known as reactive gliosis [14, 15]. It is necessary for CNS morphogenesis as it provides structural support to neurons [16] and hence it is considered as a marker of glial plasticity which controls structure, proliferation, and adhesion of astrocytes, neuron glia interactions, and CNS mechanisms. Therefore, glial cell loss may contribute to the impairment of learning and memory. Therapeutic approaches to combat human neurodegenerative diseases thus need to restore the function of both neurons and glial cells [10].

Various cellular proteins are altered upon challenge of lead induced oxidative stress and many undesired side effects of lead induced toxicity are known which include neurological [16], behavioural [17], immunological [18], renal [19], and hepatic [20] dysfunctions. We examined the expression of several proteins including HSP70, mortalin protein, immunoglobulin superfamily protein NCAM, and an intermediate filament protein GFAP to assess Ashwagandha mediated rejuvenating effects on characteristics like proliferation, adhesion, and differentiation. Lead toxicity is well known for causing oxidative stress in brain cells which can lead to damage and eventual cell death. HSP70 acts as molecular chaperon and assists in the correct folding of the target proteins and it is induced under various environmental stresses [21]. To further study the protective effect of Ashwagandha in tissues other than the brain, HSP70 expression was also studied in the peripheral organs: liver, kidney, and heart. Mortalin has been shown to regulate the homeostasis of Ca^++^ in the mitochondria that are very important for neuron functioning [22]. NCAM regulates cell adhesion and neurite outgrowth by means of homophilic binding and subsequent activation and intracellular signalling through mitogen activated protein kinase (MAPK) pathway [23]. NCAM is neuronal plasticity marker involved in cell adhesion, migration, and neurite extension [24]. The low-level lead exposure has been shown to attenuate the expression of all three major NCAM isoforms and induced reductions in neuronal growth and survival [25].

Nervous tissues have high lipid contents, thus possess high aerobic metabolic activity, which makes it more susceptible to oxidative damage [26]. C6 glioma cell line is well-established* in vitro* model system for toxicity studies due to its astroglial origin. The present study was aimed at evaluating the cytoprotective potential of Ashwagandha leaf water extract against lead induced toxicity using both* in vitro* and* in vivo *model systems. To achieve these objectives, protein and mRNA expression of markers of differentiation such as GFAP and NCAM as well as cytoprotection like HSP70 and mortalin were studied. Further protective effects of Ashwagandha leaf extract were evaluated in the brain and peripheral organs in lead intoxicated animal model system.

## 2. Material and Methods

### 2.1. Preparation of Water Extract of Ashwagandha Leaves (ASH-WEX)

The Ashwagandha leaf extract was prepared by suspending 10 g of dry leaf powder in 100 mL of double distilled water. The suspension was stirred at 45 ± 5°C overnight and filtered. The filtrate so obtained was treated as 100% water extract.

### 2.2. C6 Glioma Cell Culture and Its Treatment

C6 glioma cell line was obtained from National Centre for Cell Science (NCCS), Pune, India. The cell line was maintained on DMEM supplemented with streptomycin (100 U/mL), penicillin (100 U/mL), gentamycin (100 *μ*g/mL), and 10% (FBS) at 37°C and 5% CO_2_. For 2-(3,5 dimethylthiazol-2 yl)-4, 5 dimethyltetrazolium bromide (MTT) assay, C6 cells were seeded in 96-well plate and were given 24 hours pretreatment of 0.1% ASH-WEX followed by treatment with different concentrations (25 *μ*M–400 *μ*M) of lead nitrate for 72 hrs. 2 hr prior to the completion of the experiment, MTT (0.5 mg/mL) was added and incubated at 37°C for 2 hr. The medium was discarded and 100 *μ*L dimethylsulfoxide was added per well to dissolve the formazons. The absorbance was recorded at 550 nm. To further evaluate the effect of lead and Ashwagandha on C6 cells, the cultures were divided into four groups: control group without any treatment (C), control group with (0.1%) ASH-WEX treatment (CA), lead nitrate treatment group (LN), and combined lead nitrate and ASH-WEX treatment (LN + AS) group. 10 mM lead nitrate filter sterilized stock solution was prepared. The cells were seeded along with 0.1% ASH-WEX. Cells were allowed to grow for 24 hours followed by lead nitrate (200 *μ*M) treatment for 72 hrs.

### 2.3. Experimental Animals

The Wistar strain young male albino rats in age group 2-3 months weighing 100–150 mg were taken. All procedures for animals care were carried out strictly in accordance with the guidelines of Institutional Animal Ethical Committee. The animals were divided into three groups, namely, group C, LN, and LN + AS, each having 4-5 rats and were kept in single cage and provided water and food ad libitum. Animals of group C were kept as control (fed equal volume of vehicle), group LN was treated with lead nitrate (40 mg/kg body weight) intraperitoneally, and group LN + AS was fed orally ASH-WEX (1 gm/kg body weight) and injected lead nitrate (40 mg/kg body weight) intraperitoneally for 15 days simultaneously.

### 2.4. Immunostaining

The C6 cells were seeded in multiwell plates on polylysine coated cover slips. After the completion of treatment, the cells were washed with ice cold PBS three times for 5 minutes. Cells were fixed with chilled 4% PFA for 15 minutes. After washing with PBS for 3x5 minutes, cells were permeabilized with 0.3% PBST for 15 minutes. Blocking was carried out with 5% NGS and 1% BSA in 0.1% PBST for 2 hours at room temperature. Cells were incubated in primary antibody (prepared in blocking solution) GFAP (1 : 500, Sigma), HSP70 (1 : 1000, Sigma, clone BRM-22), mortalin (1 : 500, gift sample from Dr. Renu Wadhwa), and NCAM (1 : 500, Abcys) for 48 hours at 4°C. After washings with PBST (0.1%), cells were incubated in FITC/TRITC conjugated secondary antibody (1 : 200) for 2 hours at room temperature. Cover slips were mounted with antifading medium. The cells were observed under fluorescent microscope (Nikon E600) and images were captured and analyzed using Image-Pro Plus software, version 4.5.1.

### 2.5. Immunohistochemistry

Brains from animals of all three groups control, lead nitrate, and lead nitrate with Ashwagandha treated rats (*N* = 4-5 for each groups) were perfused transcardially with 4% paraformaldehyde in phosphate buffer saline (PBS) (0.1 M) and then cryopreserved in 20% and 30% sucrose in phosphate buffer saline each for 24 hrs at 4°C. 30 *μ*m coronal sections of brain were cut using cryostat microtome set at −20°C and sections were washed in 1X PBS (3X for 15 min). Sections were then permeabilized in 0.3% PBS-Triton X-100 (pH 7.4, 0.1 M) for 30 min. Then, sections were washed with 0.1% PBST for 15 min. After washing sections were preincubated for 1 hr at room temperature in blocking solution 5% NGS in PBS with 0.32% Triton X-100 for blocking nonspecific binding sites. The sections were then incubated in the primary antibody anti-IgG for GFAP with appropriate dilution (1 : 500) in 0.32% PBST for 48 hrs at 4°C. Sections were then washed in 0.1% PBST and incubated with fluorescent conjugated secondary antibody (anti-mouse IgG FITC diluted 1 : 200 for GFAP) in 0.3% PBST for 2 hrs. Sections were then mounted on the glass slide and covered with antifading mounting fluoromount medium for capturing fluorescent images using Nikon E600 fluorescent microscope and CoolSnap CCD camera. GFAP immunostaining was observed in hippocampus, hypothalamus, and cortex regions of brain. Quantitative image analysis for immunostaining intensity measurement was done using Image-ProPlus version 4.5.1 (Media Cybernetics, USA). Intensity of immunoreactivity and the number of GFAP positive cells were quantified in random selected fields in each section using the count/size command of Image-Pro Plus software.

### 2.6. Western Blotting

#### 2.6.1. Sample Preparation

After 72 hr of treatment, the cells were washed with PBS and were harvested with PBS-EDTA (1 mM). The pellets were homogenized for 2 minutes and the homogenate so produced was centrifuged at 10,000 rpm for 15 minutes at 4°C. Supernatant was collected and the protein was estimated using Bradford's method.

For* in vivo* studies, animals from all three groups (*n* = 3 for each group) control, lead nitrate, and lead nitrate with Ashwagandha were sacrificed by cervical dislocation and decapitated. Brain was dissected, and brain regions, hippocampus (HC), hypothalamus (HT), and cortex were separated. Different brain regions and peripheral organs such as liver, kidney, and heart were homogenized in 5 vol. of chilled homogenizing buffer containing 20 mM Tris, 150 mM NaCl, 10 mM NaF, 1 mM NaVO_4_, 0.01 mM PMSF, DTT, and 1% tritonX-100 and centrifuged for 10 min at 10,000 rpm. Protein content in supernatant was determined by the Bradford method. Each homogenate was then diluted in homogenization buffer so as to equilibrate the protein content in all the samples.

#### 2.6.2. SDS-PAGE and Chemiluminescence Detection

The SDS-PAGE electrophoresis was carried out under standard denaturing conditions at 15 mA. After electrophoresis, the resolved proteins were transferred (semidry transfer) to blot PVDF membrane Immobilon P (Millipore). The transfer was carried out at 25 V for 2 hr. After transfer, the membrane was put in nonfat protein (5% skimmed milk in 0.1% TBST) for 2 hr at room temperature. The membrane was incubated with monoclonal antibody for GFAP (1 : 2500), HSP70 (1 : 5000), and mortalin (1 : 1000) in blocking solution overnight at 4°C. Membrane was subjected to three washings with 0.1% TBST each for 5 min followed by incubation with 1 : 7000 diluted HRP conjugated goat anti-mouse IgG for 2 hr at room temperature. Enhanced chemiluminescence (ECL) was used for the detection of protein bands of interest. The developed blots were subjected to analysis by intensity measurement using Alpha Imager Software.

### 2.7. qRT-PCR

Total RNA was extracted from cells by the TRI reagent (Sigma) according to the manufacturer's instruction. The integrity of the isolated RNA was checked by nondenaturing agarose gel electrophoresis. Equal amounts of RNA were used for cDNA synthesis. cDNAs were synthesized in 20 *μ*L reactions containing 200 units M-MLV reverse transcriptase, 4 *μ*L 5X first strand buffer, 2 *μ*L DTT (0.1 M) (Invitrogen), 5 *μ*g of total RNA, 1 mM each of dNTPs (Amersham), 20 units of ribonuclease inhibitor (Sigma), and 250 ng pd(N)6 random hexamers (MBI, Fermentas).

2 *μ*L of cDNA was amplified in a 50 *μ*L PCR reaction mixture containing two units Taq polymerase, 5 *μ*L 10X PCR buffer, 1.5 *μ*L of 50 mM MgCl_2_ (Invitrogen), 1 *μ*L of 10 mM dNTP mix (Amersham), and 20pM respective primers as listed in Table 1. Cycling conditions comprised an initial denaturation of 3 min at 94°C followed by 35 cycles of amplification (at 94°C for 40 sec, 55°C for 45 sec, and 72°C for 1 min) and final elongation step at 72°C for 10 min. To control the PCR reaction components and the integrity of the RNA, 2 *μ*L of each cDNA sample was amplified separately for *β*-actin specific primer.

### 2.8. Estimation of Activities of Antioxidative Enzyme and Levels of Antioxidants

#### 2.8.1. Preparation of Sample

Animals from all three groups (*n* = 5 for each group) control, lead nitrate, and lead nitrate along with Ashwagandha were sacrificed by cervical dislocation and decapitated. Brain was dissected, and brain regions, hippocampus (HC), hypothalamus (HT), and cortex were separated. Different brain regions and peripheral organs such as liver and kidney were homogenized in 10 volume of child homogenizing buffer containing 250 mM sucrose, 12 mM Tris-HCl, and 0.1 mM DDT, at pH 7.4. Homogenates were centrifuged at 10,000 rpm and used for further estimation of antioxidative enzymes.

#### 2.8.2. Estimation of Catalase

Catalase activity was measured according to the method of Aebi [27]. The reaction mixture (1 mL) contained 0.8 mL phosphate buffer (0.2 M, pH 7.0) having 12 mM H_2_O_2 _vol. substrate, 100 *μ*L enzyme sample to make up the volume. The change in absorbance/minute was taken at 240 nm against H_2_O_2_-phosphate buffer as blank. Enzyme activity was determined as one unit of catalase equal to decomposition of 1 *μ*m of H_2_O_2_ per min at pH 7.0 at 25°C.

#### 2.8.3. Estimation of Cu-Zn SOD

Activity of superoxide dismutase (SOD) was estimated according to the method of Kono [28]. The principle involved the inhibitory effects of SOD on the reduction of nitro blue tetrazolium (NBT) dye by superoxide radicals, which are generated by the autoxidation of hydroxylamine hydrochloride. Briefly, the reaction mixture contained 1.3 mL sodium carbonate buffer (50 mM), pH 10.0, 500 *μ*L NBT (96 *μ*M), and 100 *μ*L Triton X-100 (0.6%). The reaction was initiated by addition of 100 *μ*L of hydroxylamine hydrochloride (20 mM), pH 6.0. After 2 min, 50 *μ*L enzyme sample was added and the percentage of inhibition in the rate of NBT reduction was recorded. One unit of enzyme activity was expressed as inverse of the amount of mg protein required to inhibit the reduction of NBT by 50%. The reduction of NBT was followed by an absorbance increase at 540 nm.

#### 2.8.4. Assay of Glutathione Peroxides (GSH) Content

The GSH content in the samples was determined as described by Sedlak and Lindsay [29]. 100 *μ*L of enzyme sample of all the groups (distilled water in the case of blank) was mixed with 4.4 mL of (0.01 M) EDTA and 500 *μ*L of trichloroacetic acid (50% w/v). The contents were centrifuged at 3000 g for 15 minutes. The supernatant so obtained was mixed with 50 *μ*L of 5-5′-dithiobis (2-nitrobenzoic acid) (0.01 M). The yellow color formed was read at 412 nm.

#### 2.8.5. Estimation of Lipid Peroxidation (LPx)

Method of Buege and Aust [30] was followed to measure the lipid peroxidation level. 100 *μ*L sample was incubated with 100 *μ*L each of FeSO_4_ (1 mM), ascorbic acid (1.5 mM), and Tris-HCl buffer (150 mM, pH 7.1) in a final volume made to 1 mL, made up by DDW, for 15 minutes at 37°C. The reaction was stopped by adding 1 mL of trichloroacetic acid (10% w/v). This was followed by addition of 2 mL thiobarbituric acid (0.375% w/v). After being kept in boiling water bath for 15 min, contents were cooled off and then centrifuged. The absorbance of supernatant so obtained was measured at 532 nm. The extent of lipid peroxidation was expressed as nanomoles of malondialdehyde consumed per minute at 25°C.

### 2.9. Statistical Analysis

Data of MTT, immunostaining intensity, Western blotting, and RT-PCR was analyzed statistically using Sigma Stat for Windows (version 3.5). The results were analyzed using one way ANOVA to determine the significance of the mean between the groups followed by post hoc comparison using Bonferroni test. Values of *P* ≤ 0.05 were considered significant. The means of the data are presented together with the standard error mean (SEM).

## 3. Results

### 3.1. ASH-WEX Modulates Lead Induced Morphological Changes as well as Viability in C6 Glioma Cells

**Figure 1:**
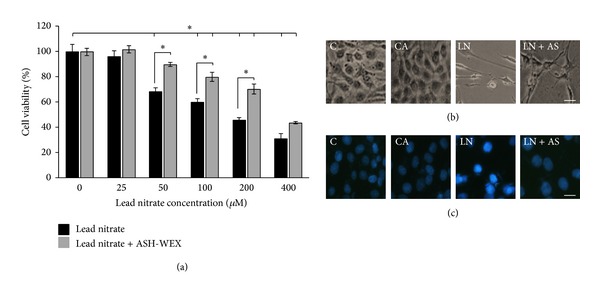
ASH-WEX protects against lead induced toxicity. (a) Relative cell viability of C6 glioma cells after lead induced toxicity as assessed by MTT assay. C6 cells were grown and pretreated with ASH-WEX (0.1%) 24 hours before exposure to lead nitrate (25–400 *μ*M) and grown for 48–72 h. Lead nitrate significantly reduced the viable cells at 50 *μ*M and higher concentrations. ASH-WEX treated cells were significantly higher in viability as compared to respective lead nitrate treated groups (in 50, 100, and 200 *μ*M). Data are representative of four different experiments and are expressed as mean ± SEM. (b) Representative phase contrast pictures of control (C), control with ASH-WEX (0.1%) pretreatment (CA), lead nitrate 200 *μ*M (LN), and lead nitrate after pretreatment with ASH-WEX (LN + AS) treatment. Images were captured using Nikon TE-2000 microscope. There was a significant difference in the cell number and morphology in lead nitrate treated cells as compared to control cells which appeared to be noramlized by ASH-WEX pretreatment in LN + AS group. (c) Cell death observed by Hoechst 33258 staining. After the cells were treated with lead nitrate and ASH-WEX, Hoechst 33258 staining was used to observe the morphological changes of cell nucleus such as condensation of chromatin and nuclear fragmentations and higher intensity of stain were found clearly in lead nitrate treated group as compared to control and LN + AS group.

There was significant decrease in cell viability as compared to control with an increase in lead nitrate concentrations (50 *μ*M onwards). The IC50 value was found to be around 200 *μ*M lead nitrate concentration, which was used for further experiments. The ASH-WEX pretreated group (LN + AS) showed significantly higher survival rate as compared to LN group (*P* < 0.001) at 50, 100, and 200 *μ*M lead concentrations. Results are presented in Figure 1(a). No such protective effect was observed at 400 *μ*M lead concentrations in the presence of ASH-WEX.

The control and 0.1% Ashwagandha pretreated cultures were found to be confluent with typical morphology of C6 cells. Most of the cells in LN group showed rounding up and altered morphology as compared to control cells. Cells grown with 0.1% Ashwagandha and treated with 200 *μ*M concentrations of lead nitrate (LN + AS) showed differentiated morphology (Figure 1(b)). No significant difference was observed in the viability of the cells in the control and CA group whereas with increase in concentration of lead nitrate the number of cells decreased. The Hoechst 33258 staining further supported the cell viability assay results as there were most of the cell nuclei in LN group appeared to be with higher intensity of stain due to chromatin condensation and nuclear fragmentation as compared to control cell nuclei. No such difference was observed in the LN + AS group nuclei (Figure 1(c)).

### 3.2. ASH-WEX Normalizes GFAP Expression, Stress Response Proteins, and NCAM* In Vitro *after Acute Lead Exposure

**Figure 2:**
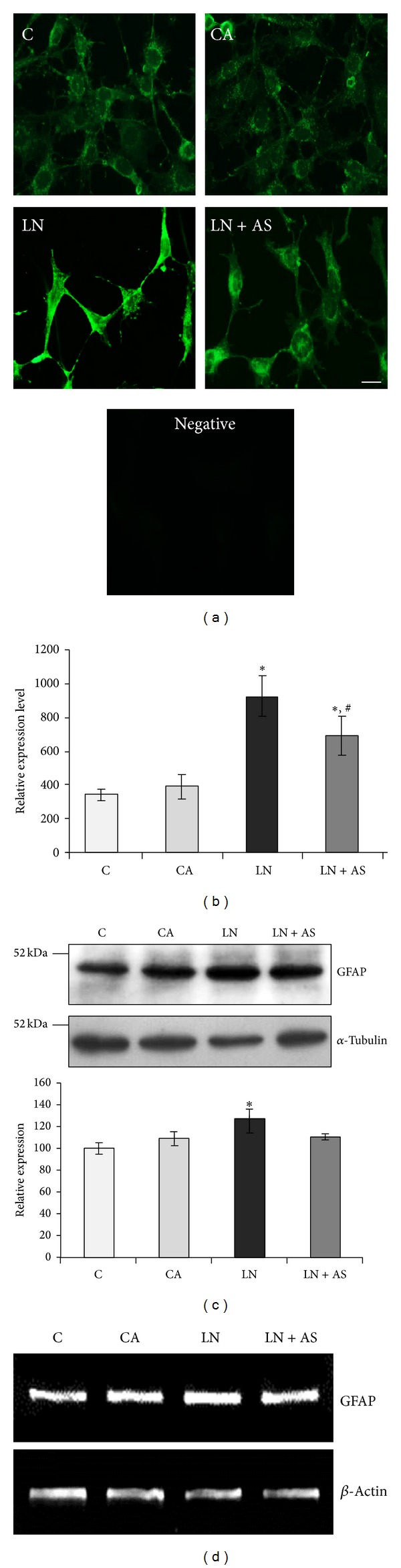
Expression of GFAP after treatment with ASH-WEX and lead nitrate. Control (C), control with ASH-WEX (0.1%) pretreatment (CA), lead nitrate 200 *μ*M (LN), and lead nitrate after pretreatment with ASH-WEX (LN + AS) cells were used for GFAP immunostaining (a) protein expression by Western blotting (c) and mRNA expression by RT-PCR (d). (b) depicts GFAP expression levels as analysed by immunostaining using intensity measurement command of Image-Pro Plus software. GFAP protein expression levels in control cells were taken as 100% to plot the histogram with relative level of expression of GFAP in C, CA, LN, and LN + AS groups. Data are calculated from three independent experiments and represented as the mean ±* * SEM. Value of *P* ≤ 0.05 was considered to be significant. *Significant difference as compared to control, ^#^significant difference between LN and LN + AS groups. *Significant difference between C and other groups and ^#^significant difference between LN and LN + AS groups.

**Figure 3:**
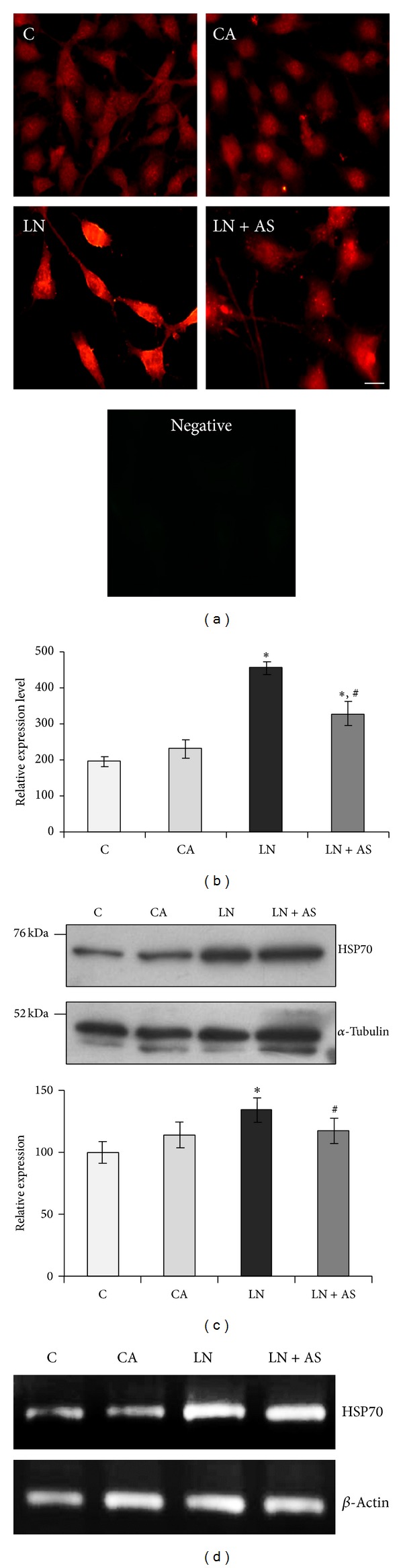
(a) Immunostaining of HSP70 in control (C), ASH-WEX (0.1%) pretreatment (CA), lead nitrate 200 *μ*M (LN), and lead nitrate after pretreatment with ASH-WEX (LN + AS) treated C6 cells. (b) Histogram depicts staining intensity measurement of HSP70 immunofluorescence indicating HSP70 expression levels in the cells from different treatment groups. (c) Representative Western blots and relative expression levels as analysed by densitometry and normalized against *α*-tubulin expression levels. The data represents mean ± SEM from three independent experiments. (d) Representative RT-PCR products of HSP70 and *β*-actin (internal control) mRNA in different treatment groups. Value of *P* ≤ 0.05 was considered to be significant. “*” represents significant difference in comparison to control; “^#^” represents significant difference between LN and LN + AS groups.

**Figure 4:**
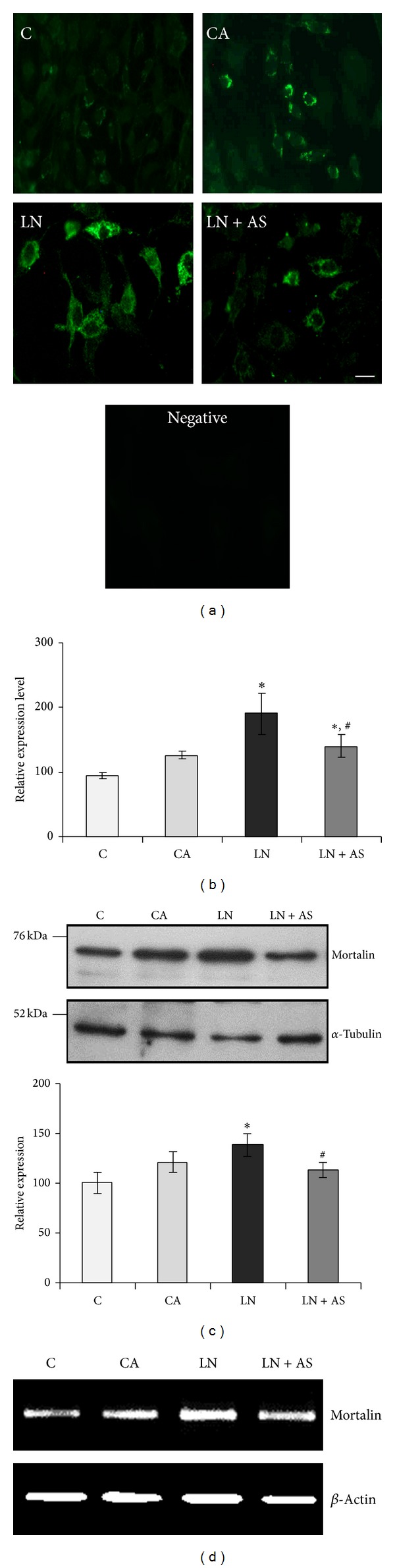
ASH-WEX pretreatment normalizes lead induced changes in mortalin expression levels. (a) Immunostaining of mortalin in C6 cells from control (C), ASH-WEX (0.1%) pretreatment (CA), lead nitrate 200 *μ*M (LN), and lead nitrate after pretreatment with ASH-WEX (LN + AS) treated groups. (b) Histogram shows expression levels of mortalin as analysed by intensity measurement using Image-Pro Plus software. (c) Representative Western blot of mortalin expression and histogram depicts relative expression levels of mortalin normalized against *α*-tubulin in C, CA, LN, and LN + AS groups as analysed by densitometry. Data are calculated from three independent experiments and represented as the mean ±* * SEM. (d) Representative RT-PCR products of mortalin and *β*-actin mRNA in all the groups under study. Mortalin expression levels were significantly lowered down by ASH-WEX pretreatment in LN + AS group as compared to LN group as shown by immunostaining, Western blotting, and RT-PCR. Value of *P* ≤ 0.05 was considered to be significant. “*” represents significant difference in comparison to control; “^#^” represents significant difference between LN and LN + AS groups.

**Figure 5:**
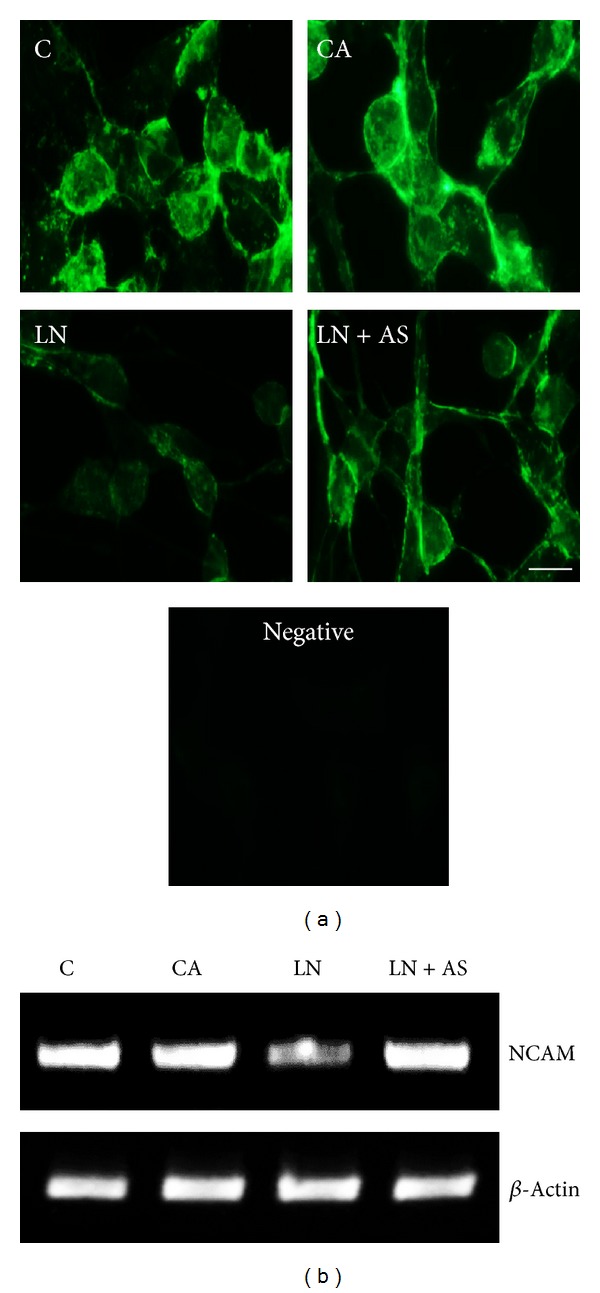
(a) NCAM protein expression levels were determined by immunostaining in control (C), ASH-WEX (0.1%) pretreatment (CA), lead nitrate (200 *μ*M, LN), and lead nitrate after pretreatment with ASH-WEX (LN + AS) treated groups. Lead treatment resulted in decreased expression of NCAM in LN group which was normalized by ASH-WEX pretreatment as shown in LN + AS group cells. These changes were also evident at transcription levels. (b) depicts representative RT-PCR products for the mRNA expression levels of NCAM and *β*-actin.

GFAP, HSP70, and mortalin protein expression was analysed using immunostaining and Western blotting. GFAP expression was lower in the control group as compared to CA group. Upon lead treatment, the expression increased significantly (Figures 2(a) and 2(b)) and was normalised upon Ashwagandha treatment in LN + AS group (*P* < 0.05). The immunostaining results were further supported by Western blotting analysis for GFAP (Figure 2(c)). RT-PCR results for GFAP mRNA also showed similar results with maximum expression in LN group and normalization upon Ashwagandha treatment (Figure 2(d)). Similarly HSP70 protein expression was enhanced in LN group upon lead treatment which was significantly reduced (*P* < 0.05) in LN + AS group as compared to LN group (Figures 3(a) and 3(b)). Western blotting and RT-PCR results for HSP70 protein further revealed similar trend as shown in Figures 3(c) and 3(d). The mitochondrial Mortalin (the mitochondrial HSP70) was perinuclear in control cells which is a characteristic pattern of transformed cells. Upon 0.1% Ashwagandha treatment, the localization of mortalin seemed to be redistributed in the cytoplasm. The mortalin protein expression was significantly increased in the LN group and was normalized in the LN + AS group. The results are shown in Figures 4(a) and 4(b). The protein expression as analysed by Western blotting further revealed significant increase in mortalin expression upon lead exposure (Figure 4(c)) which was normalized in the presence of ASH-WEX (LN + AS group). Similarly, there was increase in mortalin mRNA expression upon lead treatment in LN group. Ashwagandha treatment leads to decline in the mortalin mRNA expression in the LN + AS group as compared to LN group (Figure 4(d)). Lead nitrate treatment apparently reduced NCAM expression in the LN group as compared to control and CA groups. Ashwagandha treatment of lead exposed C6 cells showed enhanced NCAM expression in the LN + AS group as shown by immunostaining in Figure 5(a). These results were further supported by semiquantitative RT-PCR data (Figure 5(b)). The rescue of C6 cells by ASH-WEX is also apparent from the morphology of cells in LN + AS group as the cells showed long processes with distinct NCAM cell surface on both the cell bodies and processes.

### 3.3. ASH-WEX Modulates GFAP Expression in Brain Regions after Lead Exposure

**Figure 6:**
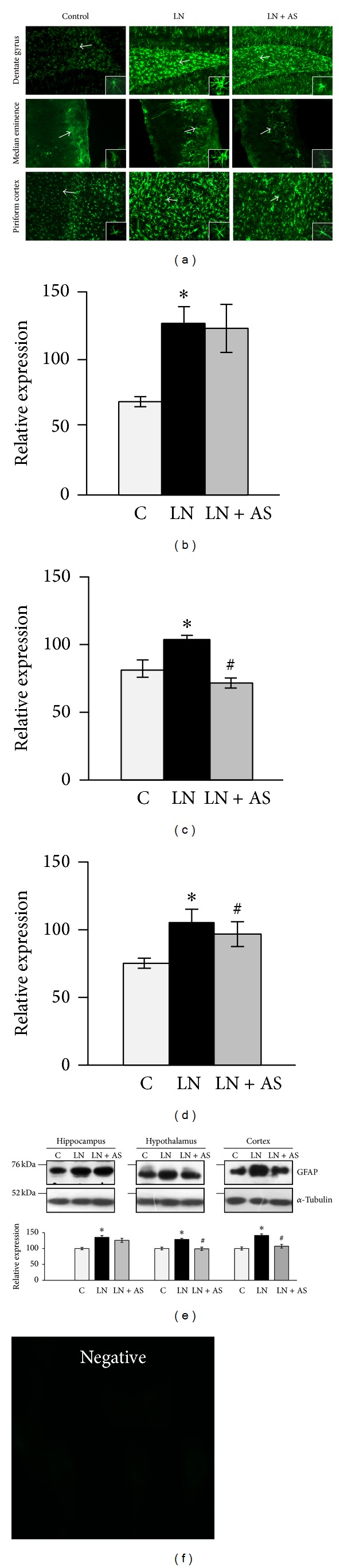
(a) Representative immunofluorescent images of 30 *μ*m thick coronal sections showing GFAP expression in dentate gyrus region of hippocampus, median eminence region of hypothalamus, and piriform cortex in vehicle control (C), lead nitrate (LN), and simultaneous lead nitrate and ASH-WEX (LN + AS) treated animal brains (*N* = 4-5). A marked increase in expression of GFAP was observed in the animals treated with lead nitrate as compared to control. The lead nitrate and ASH-WEX treated group showed normalization in expression of GFAP levels. Relative expression levels of GFAP were analysed using intensity measurement command of Image-Pro Plus software in hippocampus (b), hypothalamus (c), and cortex (d) shown as histograms. (e) Representative Western blot for GFAP and *α*-tubulin expression levels for different brain regions—hippocampus, hypothalamus, and cortex from C, LN, and LN + AS treated animals. GFAP protein expression levels were normalized against *α*-tubulin and data was plotted as histograms. Bar shows the mean ± SEM values. (f) depicts specific immunostaining for GFAP shown above as no specific signal was visible in the secondary antibody control group. *P* < 0.05 was considered significant. “*” represents significant difference between C and other groups and “^#^” represents significant difference between LN and LN + AS groups.

GFAP is excellent marker of astrocytes activation, which responds to CNS damage with reactive gliosis, induced by many neurotoxic agents. Immunohistochemical staining was done to localize the GFAP protein expression in brain regions. Lead nitrate treated group showed a considerable increase in expression of GFAP in the brain regions like hippocampus (*P* < 0.001), hypothalamus (*P* < 0.001), and piriform cortex (*P* < 0.05) (Figure 6(a)). Simultaneous lead nitrate and Ashwagandha treatment showed considerable decrease of GFAP expression in hypothalamus (*P* < 0.001, Figure 6(c)) and piriform cortex (*P* < 0.05, Figure 6(d)), but there was no statistical significant difference in hippocampus (Figure 5(b)). No specific signal was observed in the negative control immunostaining (Figure 6(f)).

GFAP expression was further confirmed by Western blotting. Lead nitrate treatment showed a significant increase in GFAP expression from different regions of brain-hippocampus (*P* < 0.01), hypothalamus (*P* < 0.01), and cortex (*P* < 0.05). Simultaneous lead nitrate and Ashwagandha treatment resulted in decrease of GFAP expression in hypothalamus (*P* < 0.001) and cortex (*P* < 0.05) regions only. Hippocampus region showed no significant decrease in GFAP level upon ASH-WEX treatment. Results are shown in Figure 6(e).

### 3.4. ASH-WEX Was Able to Normalize HSP70 Levels in Brain Regions and Peripheral Organs

**Figure 7:**
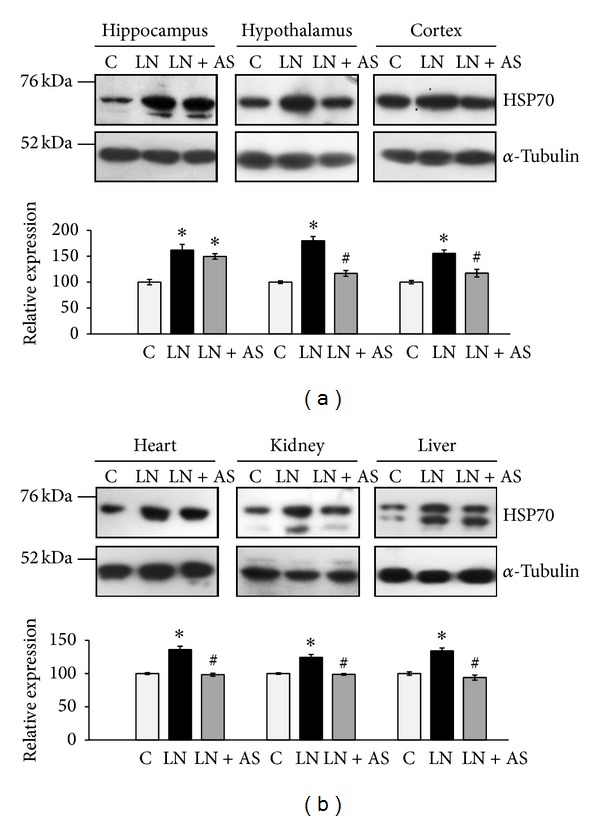
(a) Representative Western blots for HSP-70 expression and *α*-tubulin for different brain regions hippocampus, hypothalamus, and cortex from control (C), lead nitrate (LN), and lead nitrate and Ashwagandha (LN + AS) treated rats. Relative expression levels of HSP-70 normalized against *α*-tubulin were plotted as histograms. (b) Representative Western blots for HSP-70 expression and *α*-tubulin from different peripheral organs: heart, kidney, and liver from control and treatment groups. Histograms represent expression levels of HSP-70 normalized against *α*-tubulin. Data are calculated and represented as the mean ±* * SEM (*N* = 4-5). *P* < 0.05 was considered significant. “*” represents significant difference between C and other groups and “^#^” represents significant difference between LN and LN + AS groups.

HSP70 expression was analyzed in brain regions such as hippocampus, hypothalamus, and cortex. Hippocampus, hypothalamus, and cortex regions showed significant increase in HSP70 level when treated with lead nitrate alone. Treatment of ASH-WEX with lead nitrate led to a significant decrease in HSP70 levels both in hypothalamus (*P* < 0.05) and cortex (*P* < 0.001) as compared to lead nitrate treatment group. The HSP70 expression remained significantly higher in LN + AS group as compared to control and no significant change in HSP70 levels was observed as compared to LN group. In order to account for potential variation and sample loading, expression of each sample was compared to that of *α*-tubulin. Results are shown in Figure 7(a).

The HSP70 levels were also examined in peripheral organs: heart, kidney, and liver. Heart (*P* < 0.001) and kidney (*P* < 0.05) showed a marked increase in HSP70 level upon lead nitrate treatment alone. Simultaneous ASH-WEX and lead nitrate treatment showed considerable reduction in HSP70 level of heart (*P* < 0.05) and kidney (*P* < 0.01) as compared to lead nitrate treatment, thereby indicating that ASH-WEX treatment counteracts lead induced stress. Administration of lead nitrate and Ashwagandha in LN + AS group showed significantly decreased HSP70 in liver (*P* < 0.02) as compared to lead nitrate treatment. Of note, HSP70 protein bands for both constitutive and induced form can be observed in the blots. In order to account for potential variation and sample loading, expression of each sample was normalized to that of *α*-tubulin. Results are shown in Figure 7(b).

### 3.5. ASH-WEX Interferes with Antioxidant Defence Status in Acute Lead Toxicity

#### 3.5.1. Catalase Activity

A statistical significant decrease (*P* < 0.002) in the CAT activity was seen after lead nitrate treatment from all the brain regions studied. Similarly, a significant decrease was observed in CAT activity in peripheral organs kidney (*P* < 0.05) as well as increase in liver (*P* < 0.02) upon lead nitrate treatment as compared to control group. Catalase activity upon feeding of ASH-WEX with lead nitrate treatment showed significant increase (*P* < 0.05) in enzyme activity in kidney, liver cortex, and hypothalamus regions of brain with no significant changes in hippocampus region of brain as compared to LN group (Table 2).

#### 3.5.2. Superoxide Dismutase (SOD)

Lead nitrate treatment alone showed significant decrease (*P* < 0.001) in SOD activity in all regions of brain as well as in kidney and liver (*P* < 0.02) as compared to control group. Treatment of ASH-WEX with lead nitrate showed no significant change on SOD in liver and hippocampus and cortex regions of brain, while hypothalamus and kidney showed significant increase in SOD activity as compared to LN group (Table 2).

#### 3.5.3. Lipid Peroxidation (LPx)

The lead nitrate exposed rats exhibited a significant increase in LPx in hippocampus (*P* < 0.05), hypothalamus (*P* < 0.02), and cortex regions of brain as well as kidney, but liver tissue showed nonsignificant increase as compared to control. Simultaneous lead nitrate and Ash-WEX treatment (LN + AS group) showed statistical significant decrease in LPx in hippocampus and hypothalamus, respectively, and also in peripheral organs as compared to LN group (Table 2).

#### 3.5.4. Glutathione Content

Lead nitrate treatment showed slight decrease in GSH content in hippocampus and hypothalamus regions of brain as well as liver. There was no significant change in kidney glutathione content. Ashwagandha treated rats showed increase in level of GSH in all the tissues under study, but the changes were not statistically significant (Table 2).

## 4. Discussion

CNS is the principal target of the neurotoxic effects of lead; however, there are no effective treatments or interventions available to counteract it. Ashwagandha is a popular Ayurvedic plant with a variety of medicinal properties and is also widely used as a nerve tonic [3]. Ashwagandha leaf extract is a potential agent in treating oxidative damage and physiological abnormalities seen in mouse model of Parkinson's disease [31]. In the light of aforementioned CNS related beneficial properties of Ashwagandha, the present study was designed to evaluate the beneficial effects of its aqueous leaf extract against lead nitrate neurotoxicity using* in vitro* (C6 glioma cells) and further analysing it* in vivo* rat model system. 0.1% ASH-WEX was able to prevent the toxic effects of lead treatment in LN + AS group as evident by normal cell morphology and viability, providing evidence for cytoprotective role of this important Indian herb.

In the present study, the upregulation of GFAP expression was observed upon lead treatment both* in vitro* and* in vivo* systems. Lead alone induced reactive gliosis which is apparent from the significantly higher expression of GFAP in all brain regions under study. Morphological changes in astrocytes coupled with immunostaining for GFAP expression showed a marked increase in its level after lead nitrate treatment. Simultaneous Ashwagandha treatment of lead exposed cultures showed significant abatement in GFAP expression and also normalizing the morphology of astrocytes. Previous studies have reported association of increased GFAP levels and hypertrophic changes with susceptibility to toxic insult in C6 rat glioma cells [32]. The acute lead exposure is accompanied by astrocyte activation associated with the enhanced expression of GFAP as an indicator of lead induced neuronal injury [15]. Ashwagandha has been shown to antagonize the DNA damage and oxidative stress induced by lead [21]. Recently, it has been shown that Ashwagandha extract and its bioactive component Withanone was able to revert scopolamine induced changes in GFAP expression in the neuronal cells as well as animal model [33]. Normalization of GFAP expression by Ashwagandha extract in glutamate induced neurodegeneration in RA differentiated cultures has been shown in a recent study from our lab [34]. Thus, downregulation of GFAP expression by ASH-WEX treatment under both* in vitro* as well as* in vivo* conditions could be attributed to protective properties of Ashwagandha.

We further evaluated the levels of HSP70 expression after lead and Ashwagandha treatment. Our results have shown an increase in HSP70 expression in the LN treated cells in response to lead induced stress, whereas, its expression level was significantly reduced in the ASH-WEX pretreated group in C6 cells. Further* in vivo* studies suggested that Ashwagandha leaf extract treatment decapitated the expression of HSP70 significantly as compared to LN treatment group in hypothalamus and cortex regions of brain, thus debilitating the cytotoxic effects of lead nitrate* in vivo*. These palliative results suggested that Ashwagandha leaf extract has a potential cytoprotective effect on different brain regions and can curtail cytotoxicity caused due to exposure of lead. Lead exposure has been implicated in induction of prenatal and postnatal induction of HSP70 in astrocytes [35]. The antibody used in present study for HSP70 (clone BRM-22) recognises both HSP70 and HSC70. Although HSP70 is known to be cytoplasmic in control cells, it is well documented that HSP70 is present in the nucleus predominantly during S-phase and at low levels during remaining cell cycle [36]. Moreover, nuclear translocation of stress protein Hsc70 during S phase in rat C6 glioma cells has also been reported [37]. So nuclear staining of HSP70 could be attributed to its cell cycle regulatory or other roles.

Furthermore, expression of mitochondrial HSP70, mortalin, was perinuclear in the control group which shifted to pancytoplasmic in case of LN group and in LN + AS group. Mortalin is nonheat inducible molecular chaperon which is induced by different environmental stresses [38]. Mortalin has been implicated in Alzheimer's (AD) and Parkinson's (PD) diseases, with proteomic studies consistently identifying oxidatively damaged mortalin as potential biomarker [29]. Significantly higher expression of mortalin as an adaptive response has been reported as a result of Ashwagandha treatment [39]. Thus, current results showing increase in mortalin in Ashwagandha treated group indicate role of mortalin in cytoprotective mechanism induced in glial cells and elucidation of molecular mechanism(s) of these functions requires further studies.

Lead nitrate treatment caused significant increase in HSP70 expression in all these organs as shown by Western blotting, which was significantly reduced after oral feeding of the animals with Ashwagandha extract. Increased expression of HSPs in liver at both transcriptional and translational levels has been associated with the early phase of liver regeneration [40]. Previously, HSP72 (inducible form of HSP70) has been shown to be upregulated during kidney injury in rats, which partially protected human kidney proximal tubule cell lines HK-2 and HKC from triptolide-induced injury [41]. Thus, normalization of HSP 70 levels in the present study after Ashwagandha treatment* in vitro* as well as* in vivo* further confirms the protective effects of Ashwagandha against lead induced toxicity.

Another important neuronal marker NCAM was found to be downregulated in C6 glioma cells after lead exposure. The low level maternal lead exposure has been shown to decrease the expression of NCAM and its glycosylated form in hippocampus of rat pups [42, 43]. Consistent with these reports, expression of NCAM was found to decrease in lead nitrate exposed C6 cells, whereas Ashwagandha treatment was observed to augment the lead mediated downregulation of NCAM partially as shown by immunostaining and RT-PCR. A recent study on Ashwagandha treatment in C6 glioma cells has shown significant increase in expression of NCAM [39] and also increase in NCAM expression after Ashwagandha treatment has been associated with protection against glutamate induced damage in RA differentiated neuronal cultures [34].

As recent studies point to the fact that at least some of the effects may occur as a consequence of lead propensity for disrupting the delicate prooxidant/antioxidant balance that exists within mammalian cells [44], we further investigated the possible mechanism of Ashwagandha induced neuroprotection against lead toxicity by analysing the cellular antioxidant defense system. Lead exposure leads to induction of ROS production, oxidative stress, and expression of proapoptotic genes and cell death [45]. The present results have shown that Ashwagandha extract does have antioxidant properties as it resulted in a decrease of lead induced rise in lipid peroxidation in all the brain regions under study as well as peripheral organs kidney and liver. In relation to the levels of SOD, here too mostly beneficial effects were seen in the brain regions. There was a nonsignificant increase in the level of SOD in the liver and cortex while the kidney and hypothalamus SOD levels were normalized. These results are consistent with the data reported by different studies with root extracts of Ashwagandha [2, 46] where an overall increase in the level of SOD and decrease in the lipid peroxidation was observed after administration of Ashwagandha extracts. Chaudhary et al. [47] has reported Ashwagandha root extract enhancing the antiperoxidation of hepatic tissue.

In contrast to the above results, there was a consistent decrease in the levels of catalase in all the regions of the brain and peripheral organs after lead treatment which was normalized by Ashwagandha extract treatment. This is in line with the effect of the root powder and extracts which have been shown to induce an increase in the catalase activity in previous studies. Bhattacharya et al. [2] have shown an increase in catalase activity in rat brain frontal cortex and striatum after treatment with roots of Ashwagandha. Panda and Kar [46] also have reported an increase in the level of catalase after treatment with roots of Ashwagandha. Similarly, Jain et al. [48] have shown that* Withania somnifera* reverses the chronic footshock induced changes, which include an increase in SOD and lipid peroxidation and decrease in catalase activity in the rat frontal brain cortex and striatum.

Administration of Ashwagandha did not show significant change in GSH in all regions understudied and is supported by the study of Bhattacharya et al., [2], in which the administration of Ashwagandha inhibits lipid peroxidation which follows a different mechanism without modifying the glutathione system, which is a main antioxidant system. GSH, selenium containing tetrameric enzyme, is the primary low molecular-weight thiol in the cytoplasm and is a major reserve for cysteine. SOD converts O_2_^−^ into H_2_O_2_ and GSH in conjunction with the reductant NADPH that can reduce lipid peroxides, free radicals, and H_2_O_2_ [49]. So, an increase in SOD in our study would indirectly indicate lowering of the free radical levels and thus point towards the antioxidant effect of the aqueous leaf extract of Ashwagandha.

The current data of protection against lead induced cytotoxicity is also consistent with several previously reported neuroprotective activities in both* in vivo* and* in vitro* models using this important medicinal plant. Recently, we reported that water extract of Ashwagandha leaves confers protection to neuronal cells against glutamate excitotoxicity [34]. Preliminary data from our lab also showed that ASH-WEX has six different water soluble molecules which may be associated with neuroprotective activity either alone or combined [39]. The bioactive components sitoindosides VII-X and withaferin A have been shown to have neuroprotective activity by binding with cholinergic receptors [48]. Modulation of release of three neurotransmitters, that is, acetylcholine, glutamate, and serotonin by Ashwagandha has been proposed to contribute to inhibition of nNOS in extract treated stressed mice [5]. Neuroregeneration of both axons and dendrites as well as reconstruction of pre- and postsynapses in the neurons was induced by withanolide A from the Ashwagandha extract which is considered to be an important candidate for the treatment of neurodegenerative diseases [50]. Based on the present findings, it may be suggested that ASH-WEX plays neuromodulatory role to rescue the glial cells against lead toxicity by suppression of stress response and upregulation of plasticity marker proteins such as GFAP and NCAM.

## 5. Conclusion

In view of our present results, we hypothesize that water soluble extract of Ashwagandha is able to partially reverse the effect of lead induced toxicity as is shown in both* in vitro* and* in vivo* systems. Detailed mechanistic studies are required to understand the mechanisms underlying the beneficial effects of Ashwagandha and to explore the optimum dosage and duration of treatment to implement the same in clinical perspectives.

## Figures and Tables

**Table 1:** Primer sequences used for semiquantitative RT-PCR.

Number	mRNA		Primer sequence	Expected product size
1	GFAP	F	5′GGC GCT CAA TGC TGG CTT CA3′	326 bp
		R	5′TCT GCC TCC AGC CTC AGG TT3′	

2	HSP70	F	5′GAG TTC AAG CGC AAA CAC AA3′	428 bp
		R	5′CTC AGA CTT GTC GCC AAT GA3′	

3	NCAM	F	5′GCC AAG GAG AAA TCA GCG TTG GAG AGT C3′	651 bp
		R	5′ATG CTC TTC AGG GTC AAG GAG GAC ACA C3′	

4	Mortalin	F	5′CAG TCT TCT GGT GGA TTA AG3′	420 bp
		R	5′ATT AGC ACC GTC ACG TAA CAC CTC3′	

5	*β*-Actin	F	5′TCACCCACACTGTGCCCATCTACGA3′	285 bp
		R	5′CAGCGGAACCGCTCATTGCCAATGG3′	

**Table 2:** Effects of Ashwagandha water extract on lead induced oxidative damage to CAT, SOD, GSH, and LPx in rat brain regions, liver, and kidney.

Groups	Kidney	Liver	Hippocampus	Hypothalamus	Cortex
Catalase (CAT)—units/minute/g					
C	10.05 ± 1.37	3.72 ± 1.20	18.80 ± 1.95	11.47 ± 0.47	10.24 ± 1.52
LN	3.80 ± 1.68*	4.95 ± 1.68*	10.57 ± 1.83*	4.58 ± 0.84*	5.35 ± 1.20*
LN + AS	12.37 ± 4.26^#^	7.00 ± 1.71^∗#^	9.56 ± 1.07*	9.31 ± 1.17^#^	11.37 ± 1.95^#^

Cu–Zn (SOD)—units/mg					
C	13.44 ± 2.83	10.89 ± 2.64	21.25 ± 4.55	19.88 ± 3.58	13.60 ± 4.61
LN	9.21 ± 1.59	6.74 ± 1.31*	12.37 ± 1.88*	11.52 ± 1.03*	8.66 ± 2.09*
LN + AS	13.03 ± 0.09^#^	7.26 ± 1.43	12.07 ± 2.51*	22.13 ± 3.03^#^	9.86 ± 2.54*

Glutathione (GSH)—units/minute/g					
C	3.34 ± 0.09	4.11 ± 0.13	3.48 ± 0.09	3.71 ± 0.11	3.22 ± 0.39
LN	3.28 ± 0.07	3.69 ± 0.16	3.29 ± 0.05	3.16 ± 0.12	3.45 ± 0.21
LN + AS	3.56 ± 0.09	3.92 ± 0.13	3.59 ± 0.05	3.54 ± 0.11	3.89 ± 0.31

Lipid peroxidation (LPx)—nmoles MDA/mL					
C	10.43 ± 2.31	20.07 ± 1.87	4.78 ± 0.23	5.29 ± 0.22	10.76 ± 0.53
LN	23.66 ± 1.75*	22.30 ± 0.53*	13.75 ± 1.71*	12.05 ± 1.03*	18.97 ± 1.65*
LN + AS	11.42 ± 0.83^#^	14.61 ± 0.67^#^	09.82 ± 1.04^∗#^	06.39 ± 0.67^#^	14.61 ± 0.82^∗#^

Data are expressed as mean ± SEM. *P* < 0.05 was considered significant. *represents significant difference compared to control group. ^#^represents significant difference compared to lead treated animals (LN group).
